# Mechanosensory feedback loops during chronic inflammation

**DOI:** 10.3389/fcell.2023.1225677

**Published:** 2023-07-10

**Authors:** Sarbari Saha, Dafne Müller, Andrew G. Clark

**Affiliations:** ^1^ University of Stuttgart, Institute of Cell Biology and Immunology, Stuttgart, Germany; ^2^ University of Stuttgart, Stuttgart Research Center Systems Biology, Stuttgart, Germany; ^3^ University of Tübingen, Center for Personalized Medicine, Tübingen, Germany

**Keywords:** epithelial barrier, inflammation, immune cells, extracellular matrix, tissue mechanics, chronic inflammatory diseases, immuno-biophysics, immuno-mechanobiology

## Abstract

Epithelial tissues are crucial to maintaining healthy organization and compartmentalization in various organs and act as a first line of defense against infection in barrier organs such as the skin, lungs and intestine. Disruption or injury to these barriers can lead to infiltration of resident or foreign microbes, initiating local inflammation. One often overlooked aspect of this response is local changes in tissue mechanics during inflammation. In this mini-review, we summarize known molecular mechanisms linking disruption of epithelial barrier function to mechanical changes in epithelial tissues. We consider direct mechanisms, such as changes in the secretion of extracellular matrix (ECM)-modulating enzymes by immune cells as well as indirect mechanisms including local activation of fibroblasts. We discuss how these mechanical changes can modulate local immune cell activity and inflammation and perturb epithelial homeostasis, further dysregulating epithelial barrier function. We propose that this two-way relationship between loss of barrier function and altered tissue mechanics can lead to a positive feedback loop that further perpetuates inflammation. We discuss this cycle in the context of several chronic inflammatory diseases, including inflammatory bowel disease (IBD), liver disease and cancer, and we present the modulation of tissue mechanics as a new framework for combating chronic inflammation.

## Introduction

Epithelial barrier tissues maintain a tight seal between the outside environment and the inside of the body. Loss of barrier integrity leads to local activation of immune cells and fibroblasts, which can remodel local ECM networks, the major determinants of tissue mechanics. Over time, these structural and molecular changes result in tissue stiffening (Barron and Wynn, 2011; Chrysanthopoulou et al, 2014; Curaj et al, 2020). During acute inflammation, increased tissue stiffness can be beneficial for regeneration and wound healing, for example, by enhancing immune cell activity and stimulating immune cell migration and infiltration (Sridharan et al, 2019; Gaertner et al, 2022; Millán-Salanova and Vicente-Manzanares, 2022; Nalkurthi et al, 2022). However, during chronic inflammation, modifications in local ECM networks can become permanent, leading to irreversible stiffening of the tissue and culminating in fibrosis (Jeljeli et al, 2019; Velotti et al, 2020).

**FIGURE 1:**
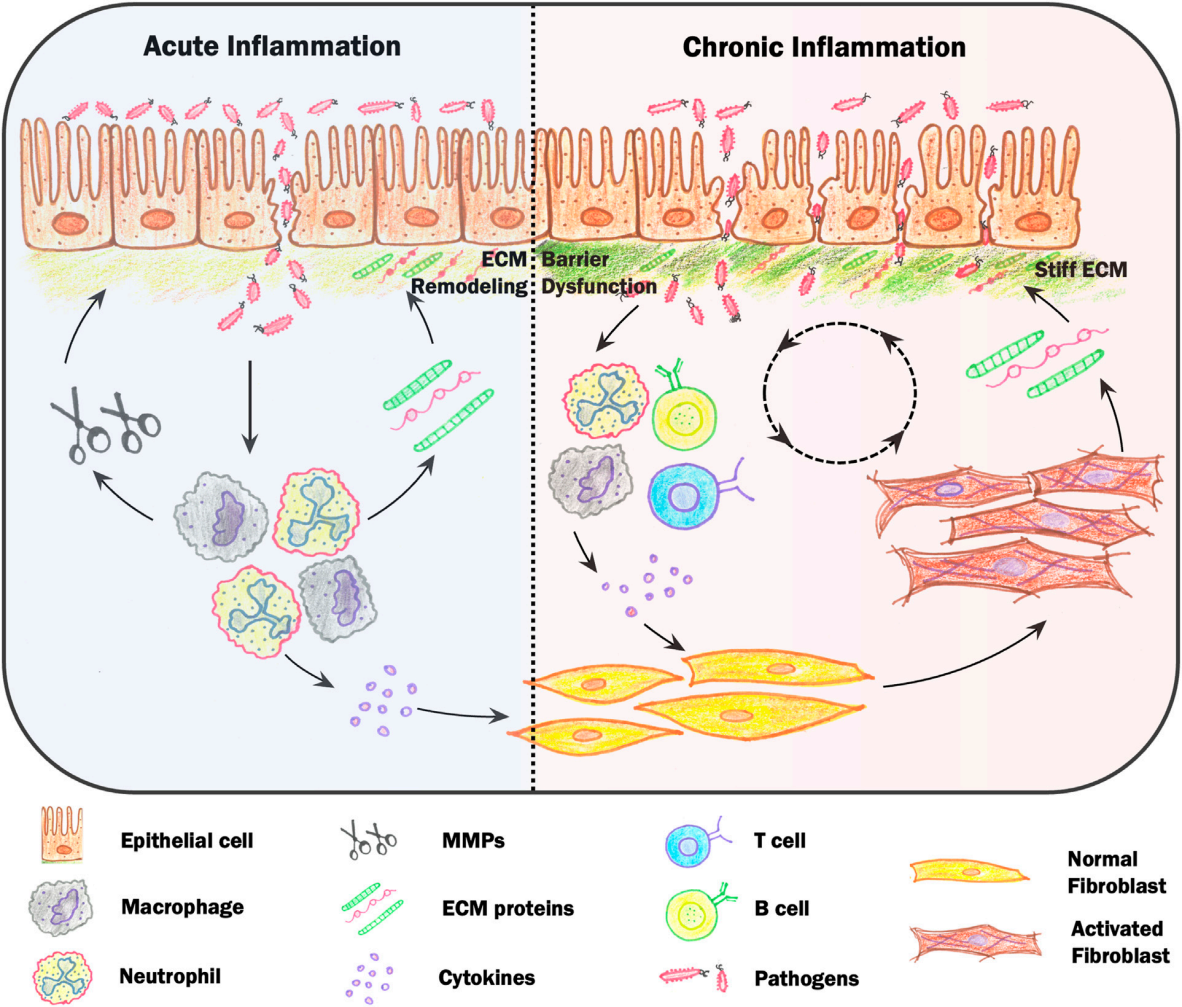
Mechanosensory feedback loops during inflammation. Loss of barrier integrity results in infiltration of microbes that initiates acute inflammation. Inflammation is led by neutrophils and macrophages, which release cytokines and chemokines and modify local extracellular matrix (ECM) structures by secretion of ECM proteins and matrix metalloproteinases (MMPs). Activated immune cells also stimulate fibroblasts, which secrete, assemble and physically remodel ECM networks, resulting in a stiffening of ECM networks. During chronic inflammation, pathologically stiff tissue can lead to over-activation of immune cells via mechanosensing pathways, resulting in increased immune cell migration and differentiation. Increases in tissue stiffness can also lead to epithelial cell depolarization, reduced cell-cell junctions and increased migration. While in the short-term this may aid in wound resealing, epithelial cells on stiff environments are less able to maintain a tight barrier, creating a positive feedback loop between increased barrier permeability and inflammation mediated by changes in tissue mechanics.

Pathologically stiff tissue can promote immune cell recruitment and activation via mechanosensing pathways, leading to increased immune cell migration and differentiation and activation of fibroblasts (Chen et al, 2020; Atcha et al, 2021; Chirivì et al, 2021; Jiang et al, 2022). Increased tissue stiffness also results in epithelial cell depolarization, reduced cell-cell junctions and increased migration (Discher et al, 2005; Aparicio-Yuste et al, 2022). While in the short-term this may aid in wound resealing, epithelial cells on stiff environments are less able to maintain a tight barrier, creating a feedback loop between increased barrier permeability and inflammation mediated by changes in tissue mechanics (Figure 1). Such mechanical feedback can ultimately disrupt organ function and presents a major risk factor for cancer development. Here, we discuss the molecular mechanisms that contribute to these feedback loops as well as pathologies where such mechanical feedback can play a role in disease progression.

## Epithelial barrier disruption leads to inflammation and local ECM remodelling

Loss of barrier integrity leads to infiltration of microbes, initiating a cascade of immune reactions whereby neutrophils and monocytes are first recruited to the site of infection (Jenne et al, 2018; Herrero-Cervera et al, 2022). These first responders not only trigger inflammation by releasing cytokines and chemokines, but also modify local extracellular matrix (ECM) structures by secretion of neutrophil elastase (NE), cathepsins, gelatinases and matrix metalloproteinases (MMPs; Delclaux et al, 1996; Ong et al, 2015; 2017; Medeiros et al, 2017). These enzymes promote the degradation of ECM components such as collagen, laminin, elastin, fibronectin and matrix bound glycoproteins (Ong et al, 2015; Xu et al, 2020). Activated neutrophils release exosomes and Neutrophil Extracellular Traps (NETs) rich in NE. NE-rich exosomes can bind to the ECM via the integrin Mac1 and degrade Collagen-I (Genschmer et al, 2019). NEs found in NETs have been shown to degrade cartilage matrix synovium, resulting in synovial joint injury (Carmona-Rivera et al, 2020). Collagenase and elastase treatment in lung tissues causes a loss and shortening of ECM fibers and decreases mechanical tissue stiffness by up to 50% (Mariano et al, 2023). In addition to degrading local ECM networks, neutrophils are also involved in tissue repair and scar formation. In response to liver injury, neutrophils physically transport existing ECM fibers to the wound site, leading to ECM accumulation at the site of damage (Fischer et al, 2022).

Similar to neutrophils, macrophages also produce and secrete various ECM-degrading enzymes (Sutherland et al, 2023). In addition, macrophages ingest and degrade ECM structures by integrin-mediated phagocytosis and receptor-mediated endocytosis (McKleroy et al, 2013; Zhao et al, 2022). On the other hand, macrophages also secrete ECM proteins including fibronectin, laminin and versican, which can help to provide a mechanical scaffold following injury and aid in the renewal of tissue architecture (Tomlin and Piccinini, 2018). Exposure to inflammatory cytokines including Transforming growth factor beta (TGF-β), Interleukin (IL)-10 and IL-13 can stimulate secretion of collagen-IV in macrophages (Schnoor et al, 2008). Differentiation of macrophages to myofibroblasts results in the production of fibrillar collagen during scar formation and ECM remodelling (Simões et al, 2020). Both macrophages and neutrophils are thus involved in degradation, production and remodeling of ECM networks and are crucial to maintaining a proper balance during homeostasis and regeneration.

When this balance is disturbed, for example, during chronic inflammation, macrophages and neutrophils can activate fibroblasts, which secrete, assemble and physically remodel ECM networks (Jeljeli et al, 2019). Culturing fibroblasts in conditioned medium from M2-like macrophages causes an increase in *Col5a1* and *Col6a1* production, leading to the production of thinner and more aligned collagen matrices. On the other hand, treating fibroblasts with hybrid M1/M2-conditioned medium results in the production of thicker, randomly oriented collagen networks. This suggests that shifting the phenotype of macrophages can promote architectural changes in the ECM via modulation of fibroblast activity (Witherel et al, 2021). In addition to molecular signals, physical cues from the microenvironment can also influence fibroblast-mediated ECM remodeling. When fibroblasts treated with M1/M2 conditioned medium are cultured on stiff substrates, they produce more aligned collagen networks compared to when they are cultured on softer hydrogels (Li and Bratlie, 2021). Fibroblasts also regulate their own activity via autocrine signaling. For example, during the inflammatory phase of myocardial infarction, activated fibroblasts produce and assemble fibrin and fibronectin and begin secreting TGF-β1, leading to a positive feedback loop of enhanced fibroblast differentiation, collagen synthesis and macrophage polarization. After reaching a stable state, a negative feedback loop is initiated, reducing TGF-β1 expression and resulting in completion of the mature scar (Curaj et al, 2020). Repeated injury and scarring can lead to a build-up of stiff fibrotic tissue that triggers fibroblasts to secrete more collagen, further driving the cycle of ECM deposition (Liu et al, 2010). Interestingly, a number of inflammatory conditions can also lead to tissue hypoxia, which, at least in tumors, can stimulate fibroblast-mediated collagen deposition and secretion of collagen-modifying enzymes including prolyl and lysyl hydroxylases (Gilkes et al, 2013). Together, these studies suggest that in various inflammatory conditions, activation of immune cells and fibroblasts leads to the reorganization of local ECM structures. During chronic inflammation, this results in a build-up of ECM and stiffening of the tissue, which can in turn stimulate immune cell activity via various mechanosensitive pathways.

## Immune cell activation by mechanosensing pathways

The innate immune system forms the first line of defense against pathogens entering the body. Leucocytes involved in the innate immune response, or myeloid cells, including macrophages, dendritic cells and mast cells, are adherent and contact-dependent, making them sensitive to changes in tissue mechanics. In particular, increased substrate stiffness, which is a result of long-term chronic inflammation, leads to increased immune cell activation and secretion of inflammatory cytokines. Lipopolysaccharide (LPS)-Activated macrophages and bone-marrow derived dendritic cells (DCs) both display enhanced production of inflammatory cytokines when cultured on mechanically stiff substrates as compared to soft hydrogels (Meli et al, 2023). DCs cultured on stiff substrates also show increased expression of glucose metabolism genes and an overall increase in their glycolytic rate, suggesting that DCs are more metabolically active on stiff substrates (Chakraborty et al, 2021). Mast cells, which are implicated in pulmonary fibrosis, are also mechanosensitive. Reseeding of healthy mast cells onto decellularized fibrotic lung tissue leads to increased degranulation and secretion of histamine and TGF-β1 compared to mast cells reseeded on healthy decellularized lung. Mechanical stretching of mast cells can produce a similar phenotype, further implicating mechanosensing in this response (Shimbori et al, 2019). The regulation of immune cell activity by increased substrate stiffness and mechanical stress is mediated by various mechanosensitive pathways including Yes-associated protein 1 (YAP) and Transcriptional coactivator with PDZ-binding motif (TAZ). High substrate stiffness leads to increased stress on the nuclear envelope, resulting in the accumulation of nuclear YAP and activation of downstream targets (Elosegui-Artola et al, 2017). In addition to YAP/TAZ signaling, stretch-activated ion channels such as piezo type mechanosensitive ion channel component 1 (PIEZO1) and Transient Receptor Potential Cation Channel Subfamily V Member 4 (TRPV4) are also involved in mechanosensing responses (reviewed in Du et al, 2022). Together, these studies suggest that immune cells involved in the innate immune response are mechanosensitive and display pro-inflammatory phenotypes in response to increased mechanical stiffness.

Cells involved in the adaptive immune response are also mechanosensitive. In order to carry out their effector functions, naïve B cells and T cells must first be activated, or “primed,” by antigen presenting cells (APCs) such as DCs. Increased stiffness of substrates designed to mimic the APC cell surface has been shown to facilitate the activation of B cells, T cells and Natural Killer (NK) cells (Judokusumo et al, 2012; Comrie et al, 2015; Meng et al, 2020). Similarly, increased stiffness of the actomyosin cortex of antigen presenting DCs enhances T cell activation (Blumenthal et al, 2020). Experiments using optical tweezers or fluid flow have demonstrated that direct application of mechanical force on T cell receptors (TCRs) can induce T cell activation (Kim et al, 2009; Li et al, 2010). Although adaptive immune cell activation is clearly mechanosensitive, it is not clear how tissue stiffness influences adaptive immune cell activity. Furthermore, B cell and T cell priming typically occurs in lymph nodes, not in the inflamed tissue. The relationship between tissue stiffness and adaptive immune priming therefore remains an open question. However, recent studies have suggested that T cell migration, along with the migration of DCs and mast cells is increased on stiff environments (Meng et al, 2020; Yu et al, 2021). This suggests that increased tissue stiffness may enhance local immune activity by stimulating both innate and adaptive immune cell migration. Increased mechanical stiffness during inflammation not only affects immune cell activity but can also have an impact on epithelial barrier integrity by directly regulating epithelial cells.

## Modulation of epithelial cell behavior by mechanical cues

The maintenance of epithelial barrier integrity is most commonly associated with tight junctions (TJs), which provide a tight seal at cell-cell boundaries and prevent the passage of materials across the epithelial layer. Recent work also suggests that adherens junctions (AJs) play a major role in epithelial integrity, either directly through mechanosensing pathways or by mediating TJ stability (Yap et al, 2018). A number of studies have demonstrated that both AJs and TJs are mechanosensitive in response to in-plane stresses arising from actomyosin contraction or external stretch, whereby moderate amounts of tensile stress led to junction reinforcement, while very high stresses cause epithelial tearing and rupture (Spadaro et al, 2017; Acharya et al, 2018; Schwayer et al, 2019). In addition to in-plane stresses, mechanosensing at cell-substrate adhesions can also affect cell-cell junction integrity. The balance between cell-cell and cell-substrate adhesions has been described as an “active wetting” phenomenon (Gonzalez-Rodriguez et al, 2012; Beaune et al, 2014; Pérez-González et al, 2019). For surfaces where cell-substrate adhesion is low, for example, very soft substrates, cell-cell adhesions dominate, leading to rounding and aggregation. This is analogous to water droplet formation on a hydrophobic surface, where liquid-substrate interactions are unfavorable and the surface tension of the droplet dominates. On substrates where cell-substrate adhesions are high, for example, on very stiff substrates, cell-substrate adhesions dominate, causing the multicellular structure to spread, or “wet” (Gonzalez-Rodriguez et al, 2012). Softer substrates therefore favor stable junctions and a tight barrier, whereas a stiff substrate favors more loosely attached cells and can also lead to dispersal into individual cells (Gonzalez-Rodriguez et al, 2012; Pérez-González et al, 2019; Ilina et al, 2020). In addition to mechanical wetting/dewetting resulting from the balance between cell-cell and cell-substrate adhesions, molecular cross-talk between different adhesion structures has also been shown to regulate cell-cell junction integrity in a substrate stiffness-dependent manner (Haas et al, 2020).

In addition to stabilization of junction proteins, efficient wound healing is a crucial aspect of tissue barrier maintenance. Wound healing requires cell migration to rapidly infiltrate the wound and actomyosin contraction to reseal the damaged area (Martin and Leibovich, 2005; Rodrigues et al, 2019). Higher substrate stiffness leads to faster wound closure mediated by increased collective migration speed and more coordinated cell movements. On stiffer substrates, actomyosin contraction slows down due to increased drag from the substrate, while crawling migration is independent of the substrate mechanics (Staddon et al, 2018; Ajeti et al, 2019). Other reports have suggested that higher stiffness can increase collective migration speeds and correlation in wound healing assays (Ng et al, 2012). It is likely that the dependence on stiffness is biphasic and highly cell-type dependent. In addition to elastic stiffness of tissues and cellular substrates, viscoelastic properties of ECM networks also influence coordinated cell movements. Crosslinking of collagen networks leads to increased network stiffness and reduces viscoelasticity, resulting in reduced collective migration (Murrell et al, 2011; Clark et al, 2022). Interestingly, changes in tissue viscoelasticity have also recently been shown to regulate collective cell behavior during development and cell invasion (Barriga and Mayor, 2019; Elosegui-Artola, 2021; Elosegui-Artola et al, 2023). Along with cell rearrangements to seal the wound, increased cell division is required to repopulate the wounded area. Substrate mechanics also regulates this process by modulating in-plane stresses generated during the resealing response, which can stimulate cell division (Zhang et al, 2003; Gudipaty et al, 2017; Donker et al, 2022). The mechanisms underlying the regulation of epithelial cell division and turnover in response to in-plane forces has been studied in several contexts (reviewed in Ragkousi and Gibson, 2014). Taken together, these studies indicate that increased substrate stiffness can perturb cell-cell junctions and cell polarity and impair the wound healing response. This suggests that the mechanical changes induced during inflammation can feed back onto epithelial cell function, resulting in further loss of barrier integrity.

## Mechanosensing feedback loops in chronic inflammatory diseases

**FIGURE 2:**
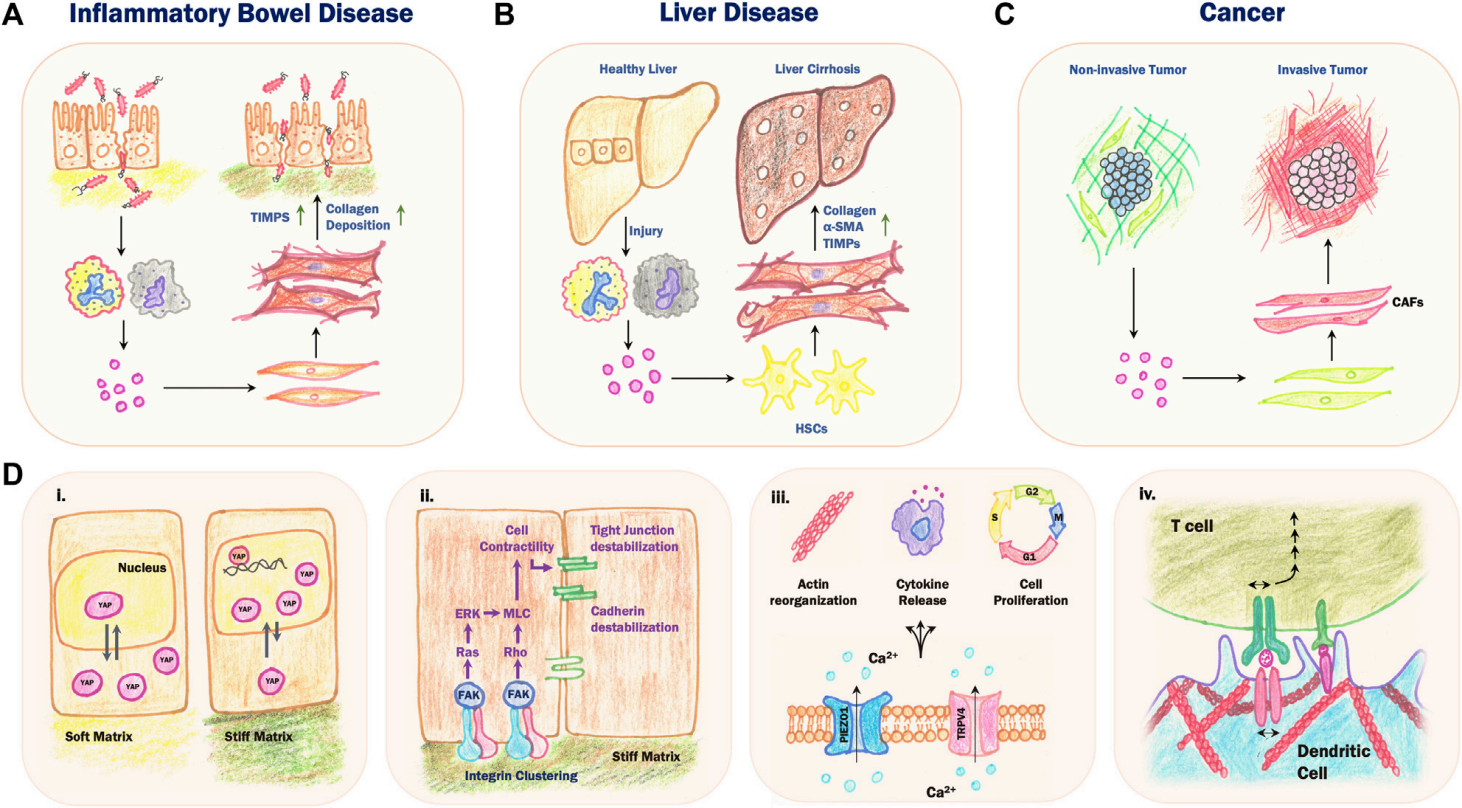
Mechanosensory feedback loops in chronic inflammatory diseases. **(A)** During Inflammatory Bowel Disease (IBD), a cycle of reduced intestinal barrier function and chronic inflammation results in increased collagen deposition and secretion of tissue inhibitors of metalloproteinases (TIMPs) by local activated fibroblasts, leading to stiffening of the underlying ECM and tissue fibrosis. **(B)** Persistent injury and inflammation in the liver results in the differentiation of hepatic stellate cells (HSCs) into activated fibroblast-like cells which secrete collagen and TIMPs and express α-SMA. This results in a replacement of the normal liver parenchyma with fibrotic scar tissue, eventually leading to fibrosis and cirrhosis. **(C)** The cross-talk between cancer cells and stromal cells leads to the activation of cancer-associated fibroblasts (CAFs), which secrete ECM proteins and matrix remodelling enzymes that contribute to increased stromal stiffness and invasion and metastasis. **(D)** Mechanochemical feedback during chronic inflammation involves various mechanosensing pathways. (i) Increased substrate stiffness results in mechanical stress on the nucleus, which inhibits nuclear export of yes-associated protein (YAP). In the nucleus, YAP acts as transcriptional coactivator to increase expression of downstream genes involved in cell proliferation and migration. (ii) High substrate stiffness leads to increased integrin clustering, which activates downstream signal transduction pathways leading to destabilization of cell-cell junctions. (iii) Mechanical stretching of the plasma membrane opens stretch-activated channels including PIEZO1 and TRPV4, leading to an influx of Ca^2+^ ions and several downstream effects including changes in actin dynamics, cytokine release and cell proliferation. (iv) Activation, or “priming”, of B cells and T cells involves heterotypic binding of membrane receptors between the B or T cell and an antigen presenting cell such as a dendritic cell. Increased stiffness of the actomyosin cortex in the dendritic cell limits mobility of the membrane receptors, resulting in increased mechanical stress on the B or T cell receptor, which enhances the activation process.

Mechanical feedback loops are likely to play a role in a number of chronic inflammatory diseases including IBD, liver disease and cancer. IBD is characterized by a cycle of increased intestinal barrier permeability and inflammation. Both immune cells and fibroblasts participate in ECM deposition and reorganization in IBD, leading to the onset of pathological tissue stiffening (Wang et al, 2022). Once tissue stiffening has begun, additional feedback mechanisms drive further tissue stiffening, leading to fibrosis and stricture formation (Figure 2A). During intestinal fibrosis, mast cell infiltration and degranulation leads to the release of large amounts of tryptase through the PAR-2/Akt/mTOR pathway, which converts fibroblasts into activated myofibroblasts. This results in deposition of collagen and fibronectin to promote intestinal fibrosis (Liu et al, 2021). Other recent work has suggested that ubiquitin-specific protease 2 (USP2), which is upregulated in intestinal myeloid cells during IBD and mouse models of colitis, increases the expression of collagen and alpha smooth muscle actin (αSMA), leading to further ECM remodeling and tissue stiffening (An et al, 2022). Collagen-I deposition in the intestine also activates the YAP/TAZ pathway in epithelial cells through Fak/Src signaling to initiate a regenerative cascade to induce a fetal-like state in the colonic epithelium, where cells become more motile and prone to reorganization compared to homeostatic conditions (Yui et al, 2018). Downstream effects of YAP/TAZ also induce the secretion of IL-33 and IL-18 and lead to cytoskeletal re-organization (Kobayashi et al, 2022). Together, these studies suggest that mechanical reorganization of ECM networks during IBD can drive further tissue stiffening, prolonged inflammation and reduced barrier function.

The liver is also exposed to various external stresses arising from dietary factors, exposure to gut microbe metabolites and alcohol and drug use, leading to tissue damage and inflammation (Lang and Schnabl, 2020; Yahoo et al, 2023). In the case of persistent inflammation, increased accumulation of ECM results in the replacement of healthy liver parenchyma with fibrotic scar tissue, further driving mechanosensitive feedback pathways (Figure 2B; Dhar et al, 2020). In mouse models of liver fibrosis, excess deposition of collagen and fibronectin along with accumulation of αSMA-expressing myofibroblasts leads to cirrhosis and increased expression of ECM genes, which correlates with poor patient prognosis (Wu et al, 2021). During this process, hepatic stellate cells (HSCs) transdifferentiate into fibroblast-like cells that express αSMA and secrete ECM components such as collagen-I and-III, fibronectin and laminin, contributing to the development of fibrosis (Friedman, 2008). HSCs also produce MMPs and Tissue Inhibitors of Metalloproteinases (TIMPs) which are the major drivers of ECM remodelling during hepatic fibrosis (Duarte et al, 2015). Chronic overexpression of TIMPs prevents normal collagen remodeling, leading to an increased collagen build-up that drives liver fibrosis (Benyon and Arthur, 2021). The resulting altered biomechanical environment can also drive liver tumorigenesis by activation of integrin-β1 and focal adhesion kinase, leading to increased cell proliferation (Schrader et al, 2011).

Chronic inflammation is a risk factor for tumorigenesis and cancer not only in the liver, but also in other tissues. The evolution of the tumor microenvironment shares many similarities with chronic inflammation, and tumors have been notably characterized as “wounds that never heal” (Dvorak, 1986; Hua and Bergers, 2019). One prominent feature of tumor progression is the cross-talk between tumor cell behavior and the increased stiffening of connective tissue surrounding the tumor (the “stroma”; Figure 2C). High stromal stiffness can lead to increased cytoskeletal activity and migration, reduced polarity and epithelial-mesenchymal transition (EMT; Clark and Vignjevic, 2015). Changes in stromal network architecture and mechanics are mediated primarily by cancer-associated fibroblasts (CAFs), which share many common features with activated fibroblasts during chronic inflammation. CAFs display increased secretion of cytokines, growth factors and matrix remodeling enzymes as well as increased mechanical force production (Sahai et al, 2020). Together, these factors drive changes in ECM organization that contribute to increased stromal stiffness, tumor invasion and metastasis. In addition, CAFs secrete proteases that cleave and activate ECM-bound cytokines and cell adhesion molecules, promoting increased migration of cancer cells and EMT (Fiori et al, 2019). The mechanical properties of the tumor stroma are also thought to contribute to immune escape mechanisms during cancer and could interfere with cancer immunotherapy (Denton et al, 2018; Ollauri-Ibáñez et al, 2021). Together, these studies suggest that similar to chronic inflammatory diseases, mechanosensory feedback loops can drive local tissue stiffening and cancer progression.

## Conclusion and outlook

Disruption of epithelial barrier tissues leads to local inflammation and activation of immune cells and fibroblasts that modify local ECM structures. Repeated injury or chronic inflammation can lead to permanent ECM remodeling and tissue stiffening, which can further exacerbate inflammation, excess fibroblast activity and barrier disruption via various mechanosensing pathways (Figure 2D). Altered tissue mechanics represents a common and general feature of chronic inflammatory diseases, despite differences in the molecular profiles of these pathologies. Future translational studies aimed at modulating tissue mechanics therefore have the potential to identify exciting new therapeutic approaches with broad applications from chronic inflammation to cancer.
